# Utilization of complementary and traditional medicine practitioners among middle-aged and older adults in India: results of a national survey in 2017–2018

**DOI:** 10.1186/s12906-021-03432-w

**Published:** 2021-10-15

**Authors:** Supa Pengpid, Karl Peltzer

**Affiliations:** 1 ASEAN Institute for Health Development, Mahidol University, Salaya, Phutthamonthon, Nakhon Pathom, Thailand; 2 Department of Research Administration and Development, University of Limpopo, Turfloop, South Africa; 3 Department of Psychology, College of Medical and Health Science, Asia University, Taichung, Taiwan

**Keywords:** Utilization, AYUSH, Traditional healer, India

## Abstract

**Background:**

Lack of information exists about the use of traditional and complementary medicine (TCM) use among middle-aged and older adults in India, which led to studying the estimates of past-12-month Ayurveda/Yoga/Naturopathy/Unani/Siddha/Homeopathy (AYUSH) practitioner and traditional health practitioner (THP) utilization in India.

**Methods:**

The study included 72,262 individuals (45 years and older) from the cross-sectional 2017–2018 Longitudinal Ageing Study in India (LASI) Wave 1.

**Results:**

The prevalence of past 12-month AYUSH practitioner utilization was 6.5%, THP use 7.0%, and AYUSH or THP use 13.0%. The rate of AYUSH practitioner utilization was determined by older age (≥60 years) (Adjusted Odds Ratio-AOR: 1.20, 95% Confidence Interval-CI: 1.07–1.34), having pain (AOR: 1.48, 95% CI: 1.29–1.69), any bone or joint diseases (AOR: 1.57, 95% CI: 1.35–1.82), current tobacco use (AOR: 1.30, 95% CI: 1.12–1.50), male sex (AOR: 0.76, 95% CI: 0.68–0.85), high subjective socioeconomic status (AOR: 0.72, 95% CI: 0.60–0.87), urban residence (AOR: 0.71, 95% CI: 0.57–0.88), diabetes (AOR: 0.66, 95% CI: 0.55–0.81), chronic heart disease (AOR: 0.52, 95% CI: 0.37–0.73), and having a health insurance cover (AOR: 0.36, 95% CI: 0.30–0.44). The rate of THP utilization was determined by depressive symptoms (AOR: 1.17, 95% CI: 1.01–1.35), sleep problems (AOR: 1.28, 95% CI: 1.08–1.51), having pain (AOR: 1.82, 95% CI: 1.55–2.15), current tobacco use (AOR: 1.35, 95% CI: 1.22–1.51), having health insurance cover (AOR: 0.41, 95% CI: 0.33–0.51), hypertension (AOR: 0.82, 95% CI: 0.71–0.95), diabetes (AOR: 0.50, 95% CI: 0.39–0.65), urban residence (AOR: 0.25, 95% CI: 0.19–0.34), and high subjective socioeconomic status (AOR: 0.70, 95% CI: 0.58–0.85).

**Conclusion:**

A moderate prevalence of AYUSH practitioner and THP use among middle-aged and older adults in India was found and several factors associated with AYUSH practitioner and THP use were identified.

## Background

Indian systems of medicine recognize AYUSH (Ayurveda, Yoga, Naturopathy, Unani, Siddha and Homeopathy) representing the tradition of codified, textual health knowledge systems other than the modern [1, 2]. Apart from these systems, there are large number of traditional health practitioners (THP) who fall under Local Health Traditions (LHT) representing the healing practices and knowledge following an oral tradition of learning and passing on of the knowledge [2, 3].

In Ayurveda, “treatment of the disease consists in avoiding causative factors responsible for disequilibrium of the body matrix or of any of its constituent parts through the use of Panchkarma procedures, medicines, suitable diet, activity and regimen for restoring the balance and strengthening the body mechanisms to prevent or minimize re-occurrence of the disease.” [4]. Yoga is a “discipline to improve or develop one’s inherent power in a balanced manner. It offers the means to attain complete self-realization. The literal meaning of the Sanskrit word Yoga is ‘Yoke’. Yoga can therefore be defined as a means of uniting the individual spirit with the universal spirit of God. According to Maharishi Patanjali, Yoga is the suppression of modifications of the mind.” [5].

Naturopathy is a “drugless non-invasive rational and evidence-based system of medicine imparting treatments with natural elements based on the theory of vitality, theory of toxemia, theory of self-healing capacity of the body and the principles of healthy living. The applied aspects of Naturopathy include mainly fasting and diet supported by treatments with natural elements which include Hydrotherapy, Chromotherapy, Mud Therapy, Manipulative therapy, Electrotherapy, Acupuncture, Magnetotherapy, Physiotherapy, Exercise & Yoga Therapy.” [6] Unani medicine has the following main types of treatment: “Regimental therapy is special technique/physical methods of treatment to improve the constitution of body by removing waste materials and improving the defence mechanism of the body and protect health. In other words, these are the best known ‘detoxification methods’, dietotherapy by regulating the quality and quantity of food several ailments are treated successfully, pharmacotherapy, which involves the use of naturally occurring drugs, mostly herbal, and minor surgery.” [7].

The Siddha System is “effective in treating chronic cases of liver, skin diseases especially Psoriasis, rheumatic problems, anaemia, prostate enlargement, bleeding piles and peptic ulcer. The Siddha Medicines which contain mercury, silver, arsenic, lead and sulphur have been found to be effective in treating certain infectious diseases including venereal diseases.” [8]. ‘Homoeopathy’ is “derived from two Greek words, *homoi* meaning similar and *pathos* meaning suffering. Homoeopathy simply means treating diseases with remedies, prescribed in minute doses, which are capable of producing symptoms similar to the disease when taken by healthy people. It is based on the natural law of healing- ‘similia similibus curantur’ which means ‘likes are cured by likes’.” [9].

Traditional health practitioners (THP) who fall under LHT are non-institutionally qualified practitioners and are a very heterogeneous group, including herbalists, spiritual or faith healers, snake bite healers, bone setters, jaundice healers, traditional birth attendants, poison healers, etc. [2, 10, 11]. While the AYUSH systems of medicine have gained official recognition, with a government AYUSH ministry, in subservience to biomedicine, as distinct from THP or LHT that all outside the frameworks of biomedicine and AYUSH [11–13]. National policy on medical pluralism in India encourages the mainstreaming of AYUSH systems and the revitalization of LHT [2, 14]. In India, there were 785,185 registered AYUSH practitioners (60.9% Ayurveda, 31.4% Homeopathy, 6.5% Unani, 0.9% Siddha, and 0.2% Naturopathy) in 2010 [15]. In all, it is estimated that “more than 1.5 million practitioners are using the traditional medicinal system for health care in India.” [16]. On the other hand, there are about 1.5 million THP in India [10].

Few studies have investigated the utilization of AYUSH and even fewer studied the utilization of THP in large population-based surveys in India. In a national Consumer Expenditure Survey in India in 2011–2012, 28.4% of Indian households used the traditional medical systems [17]. In a nationally representative health survey in India in 2014, the outpatient utilization of AYUSH care in the past 2 weeks for all ages was 6.9% and for persons 60 years and older 7.7% [18]. In a national study among persons predominantly 50 years and older in India in 2007, 19.0% reported THP use in the previous 12 months [19]. In a small study (*N* = 250) among older adults (≥60 years) in Maharashtra, India, 3.6% sought care from AYUSH and/or THP [20]. However, these studies did not report on the utilization of both AYUSH and THP differentially among middle-aged and older adults in general or for specific illness conditions of the last outpatient visit. To address these shortcomings, we analysed more recent national data on the utilization of both AYUSH and THP among middle-aged and older adults in general and for specific illness conditions of the last outpatient visit in India in 2017–2018.

Globally, the prevalence of the utilization (past 12 months) of traditional and complementary medicine (TCM) provider was 21.1% in nine high-income countries [21], and in a 32-country study 26.4% [22]. Determinants of TCM or TCM provider use may include “sociodemographic factors (female gender, age, lower socioeconomic status, and rural residence) and health-related factors (chronic disease, poor physical and mental health, inadequate health care access, and satisfaction with TCM services)” [22]. In India, traditional and AYUSH systems of medicine have a higher rate of utilization in poorer population groups [20], in particular geographical areas (states) [20], in the tribal population [12], among patients with chronic diseases and also for treating skin-related and musculoskeletal ailments [18]. This study aimed to estimate the prevalence and correlates of the utilization of both AYUSH practitioner and THP among middle-aged and older adults in a national population survey in India.

## Methods

### Sample and procedures

Cross-sectional data from the nationally representative Longitudinal Ageing Study in India (LASI) Wave 1, 2017–2018 were analyzed (the overall household response rate is 96%, and the overall individual response rate is 87%) [23]. Face-to-face interview, physical measurement and biomarker data were collected by male and female trained research teams from individuals aged 45 and above and their spouses, regardless of age, in a household survey from 35 states and union territories of India (excluding Sikkim). Field teams input responses directly into a laptop computer. Details of the sampling strategy have been described elsewhere [23]. Briefly, “LASI adopted a multistage stratified area probability cluster sampling design to arrive at the eventual units of observation: older adults aged 45 and above and their spouses irrespective of age. India is a union comprising 30 states and 6 union territories. Within each state, LASI Wave 1 adopted a three-stage sampling design in rural areas and a four-stage sampling design in urban areas. In each state/union territories, the first stage involved the selection of Primary Sampling Units (PSUs), that is, sub-districts (Tehsils/Talukas), and the second stage involved the selection of villages in rural areas and wards in urban areas in the selected PSUs. In rural areas, households were selected from selected villages in the third stage. However, sampling in urban areas involved an additional stage. Specifically, in the third stage, one Census Enumeration Block (CEB) was randomly selected in each in urban area. In the fourth stage, households were selected from this CEB. The goal was to select a representative sample in each stage of sample selection.” [23]. The study was approved by the Indian Council of Medical Research (ICMR) Ethics Committee and written informed consent was obtained from participants [23].

### Measures

#### Outcome measure

*Utilization of TCM practitioners* was assessed with two questions*,* 1) “In the past 12 months, have you consulted any AYUSH practitioner (Ayurveda /Yoga/Naturopathy/Unani/Siddha /Homeopathy)? (Yes, No)” and 2) “In the past 12 months, have you consulted any Traditional /Folk healers (tribal medicine/bhopa/jhaad-fook/black magic)? (Yes, No).” [23]. *Bhopa* are priest-singers of the folk deities in the state of Rajasthan, India [24].

*Reasons for most recent outpatient visit* was assessed with two questions, 1) “Which type of health care provider did you visit, or came to visit you, most recently in the past 12 months?” Response options were grouped into three groups, 1) biomedical practitioner (doctor, dentist, nurse/midwife, physiotherapist, and pharmacist), 2) AYUSH practitioners and 3) traditional healer; and 2) “What was the main reason of your most recent outpatient visit?” [23]. Response options included a list of 26 conditions, such as cancer (see Table 3).

*Treatment outcome* was assessed with three questions, 1) “What was the outcome of your most recent visit to the health care provider?” (Responses ranged from 1 = got much better to 5 = got much worse); 2) “Overall, how satisfied were you with health care you received at this visit?” (Responses ranged from 1 = very satisfied to 5 = very dissatisfied); and 3) “In your last visit how much you or your household pay for health care provider’s fees?” (Rupees) [23].

### Exposure variables

*Sociodemographic information* included age (years), sex (male, female), residence (rural, urban) and subjective socioeconomic status. The latter was sourced from the item, “Please imagine a ten-step ladder, where at the bottom are the people who are the worst off – who have the least money, least education, and the worst jobs or no jobs, and at the top of the ladder are the people who are the best off – those who have the most money, most education, and best jobs. Please indicate the number given (1-10) on the rung on the ladder where you would place yourself” [23]. Socioeconomic steps 1 to 3 were classified as poor, 4–5 as medium, and 5–10 as high socioeconomic status.

*Sleep problems* were assessed with four questions: 1) “How often do you have trouble falling asleep?” 2) “How often do you have trouble with waking up during the night?” 3) “How often do you have trouble with waking up too early and not being able to fall asleep again?” 4) “How often did you feel unrested during the day, no matter how many hours of sleep you had?” Responses options were “never, rarely (1-2 nights per week), occasionally (3-4 nights per week), and frequently (5 or more nights per week)” [23]. Sleep problems were coded as “frequently” for any of the four symptoms as one [25].

*Depressive symptoms* were assessed with a modified Centre for Epidemiological Studies Depression Scale (CES-D-10) [26]. The 10 items included seven negative symptoms (trouble concentrating, feeling depressed, low energy, fear of something, feeling alone, bothered by things, and everything is an effort), and three positive symptoms (feeling happy, hopeful, and satisfied). Response options included rarely or never (< 1 day), sometimes (1 or 2 days), often (3 or 4 days), and most or all of the time (5–7 days) in a week prior to the interview. For negative symptoms, rarely or never (< 1 day), and sometimes (1 or 2 days) were scored zero, and often (3 or 4 days) and most or all of the time (5–7 days) categories were scored one. Scoring was reversed for positive symptoms. The overall score ranges from zero to 10 and scores of four or more were indicative for depressive symptoms [27]. The Cronbach α of the CES-D-10 in this study was 0.79.

*Chronic conditions* were assessed with the question, “Has any health professional ever told you that you have…?”: 1) **“**Hypertension or high blood pressure (Yes/No); 2) Diabetes or high blood sugar; 3) Cancer or malignant tumor; 4) Chronic lung disease such as asthma, chronic obstructive pulmonary disease/Chronic bronchitis or other chronic lung problems; 5) Chronic heart diseases such as Coronary heart disease (heart attack or Myocardial Infarction), congestive heart failure, or other chronic heart problems; 6) Stroke; 7) Arthritis or rheumatism, Osteoporosis or other bone/joint diseases; and 8) Any neurological, or psychiatric problems such as depression, Alzheimer’s/Dementia, unipolar/bipolar disorders, convulsions, Parkinson’s etc. (Yes/No)” [23].

*Symptom-based pain* was defined as troubled by pain and required some form of medication or treatment for relief of pain [23].

*Current tobacco use* was sourced from two items, 1) “Do you currently smoke any tobacco products (cigarettes, bidis, cigars, hookah, cheroot, etc.)? and 2) Do you use smokeless tobacco (such as chewing tobacco, *gutka*, pan masala, etc.)?” [23].

Health care insurance was assessed with the item, “Are you covered by health insurance? (Yes/No)” [23].

### Data analysis

Due to the multistage stratified area probability cluster sampling design survey weights are applied at the household and individual levels. The purpose of the weights is to compensate for unequal selection probabilities at various levels of selection and to compensate for non-response [23]. Descriptive statistics were used to describe sociodemographic, health, and traditional and complementary medicine variables. Unadjusted and multivariable logistic regression was used to estimate the prevalence of past 12-month utilization of AYUSH and THP. Results from the logistic regression analyses are presented as odds ratios (ORs) with 95% confidence intervals (CIs) using Taylor linearization methods. Utilization data had ≤1.0% missing data and other variables had under 3.5% of the data missing, which were excluded from the analyses. No multi-collinearity was detected. *P* < 0.05 was considered significant. All statistical operations were conducted with STATA software version 15.0 (Stata Corporation, College Station, TX, USA), taking the multistage sample design into account.

## Results

### Sample characteristics

The study sample included 72,262 persons aged 45 years and older (female spouse, any age) from India. Table 1 describes the sample characteristics. The prevalence of past 12-month AYUSH practitioner utilization was 6.5%, traditional healer consultation 7.0%, and AYUSH or traditional health practitioner 13.0% (see Table 1).

**Table 1:** Sample characteristics

Variable	Sample	AYUSH practitioner	Traditional /Folk healers
	*N* (%)	%	%
All	72,262	6.5	7.0
Age (years)			
45–59 (female spouse: any age)	40,785 (54.1)	6.0	6.6
≥ 60	31,477 (45.9)	7.2	7.5
Sex			
Female	41,685 (58.0)	6.9	7.1
Male	30,577 (42.0)	6.0	6.8
Subjective socioeconomic status			
Low (1–3)	23,625 (37.2)	7.5	9.3
Medium (4–5)	28,380 (38.7)	6.7	6.4
High (6–10)	18,134 (24.1)	4.8	4.9
Residence			
Rural	46,539 (68.2)	7.4	9.4
Urban	25,723 (31.8)	4.6	1.7
Hypertension	19,878 (26.4)	5.8	5.1
Diabetes	8716 (11.6)	4.0	6.9
Chronic lung disease	3902 (6.3)	7.3	6.0
Cancer	472 (0.6)	8.2	7.9
Chronic heart disease	2441 (3.6)	3.4	3.8
Stroke	1220 (1.8)	7.2	5.3
Any bone or joint diseases	10,163 (15.7)	9.2	7.1
Neurological or psychiatric problem	1569 (2.4)	6.1	5.8
Depressive symptoms	17,650 (27.6)	7.1	8.6
Sleep problems	8367 (12.7)	6.6	10.0
Symptom-based pain	19,145 (27.7	8.9	9.9
Current tobacco use	21,071 (30.4)	7.6	9.3
Has health insurance	16,558 (20.7)	3.0	3.5

AYUSH practitioner (Ayurveda/unani/siddha/homeopathy); Traditional/Folk healers (tribal medicine/bhopa/jhaad-fook/black magic)

### Utilization of AYUSH and traditional health practitioner by state

The highest prevalence of AYUSH practitioner utilization in the past 12 months was in Uttar Pradesh (18.7%), followed by Maharashtra (13.8%), Bihar (8.1%), Kerala (7.7%) and West Bengal 6.9%), while the highest traditional health practitioner utilization was in Bihar (24.3%), followed by West Bengal (16.1%), Uttar Pradesh (15.3%), Jharkhand (9.7%), and Madhya Pradesh (7.9%) (see Table 2).

**Table 2:** Utilization of AYUSH and traditional health practitioner in the past 12 months by state (*N* = 72,262)

State in India	AYUSH practitioner	Traditional/folk healer
	%	%
Jammu & Kashmir	1.2	0.2
Himachal Pradesh	5.0	1.5
Punjab	2.3	5.5
Chandigarh	0.9	0.8
Uttarakhand	2.4	3.2
Haryana	3.9	2.3
Delhi	1.1	0.0
Rajasthan	1.0	1.2
Uttar Pradesh	18.7	15.3
Bihar	8.1	24.3
Arunachal Pradesh	0.5	1.2
Nagaland	0.1	0.2
Manipur	0.9	3.2
Mizoram	0.2	0.2
Tripura	2.2	0.8
Meghalaya	0.4	1.2
Assam	0.9	0.2
West Bengal	6.9	16.1
Jharkhand	1.9	9.7
Odisha	1.6	0.4
Chhattisgarh	1.7	6.6
Madhya Pradesh	4.4	7.9
Gujarat	4.7	0.5
Daman & Diu	1.5	0.1
Dadra & Nagar Haveli	1.6	1.7
Maharashtra	13.8	0.3
Andhra Pradesh	0.8	4.0
Karnataka	6.3	1.9
Goa	2.1	0.3
Lakshadweep	0.9	0.0
Kerala	7.7	0.7
Tamil Nadu	0.9	0.1
Puducherry	1.8	0.0
Andaman & Nicobar Islands	1.4	0.1
Telangana	0.2	6.0

### Main indication of most recent outpatient visit

The distribution of the most recent outpatient visit (*N* = 37,852) was 83.8% biomedical, 8.2% AYUSH and 8.0% traditional healer. For the traditional health and AYUSH practitioner, the most common reason for the most recent outpatient visit was fever or pyrexia of unknown cause (37.6%), followed by chronic pain (17.7%), generalized pain (12.7%), and high blood pressure (4.6%). The rank order of the most common reason for the most recent visit to a biomedical practitioner was the same as with the traditional health and AYUSH practitioner. The prevalence of fever or pyrexia of unknown cause and chronic pain was lower and high blood pressure was higher among biomedical than traditional health and AYUSH practitioner patients (see Table 3).

**Table 3:** Main indication of most recent outpatient visit (N = 37,852)

Main indication for most recent outpatient visit	Biomedical (*N* = 33,615)	AYUSH (*N* = 2120)	Traditional healer (*N* = 2117)	AYUSH and/or traditional healer (*N* = 4237)
	%	%	%	%
Fever/pyrexia of unknown cause	27.2	33.1	42.1	37.6
Chronic pain in your joints/arthritis/rheumatism/osteoporosis (joints, back, neck, muscle)	13.9	19.7	15.7	17.7
Generalized pain (stomach, headache, migraine, or other nonspecific pain)	10.0	13.7	11.8	12.7
High blood pressure (hypertension)	9.3	4.3	4.9	4.6
Problems with your breathing	3.5	3.5	2.2	2.9
Gastroenteritis or other diarrheal illness	2.9	3.2	2.1	2.6
Problems with your mouth/teeth/gum/lips/swallowing/throat	3.1	2.1	2.5	2.3
Skin diseases	2.0	2.9	1.5	2.2
Gastritis/acidity	1.6	1.6	2.3	2.0
Injury/accident (non-occupational)	2.7	1.6	2.3	1.9
Liver diseases (hepatitis, alcoholic liver disease, cirrhosis)	1.1	1.2	2.0	1.6
Diabetes or related complications	6.6	1.8	0.9	1.3
Malaria	1.2	1.1	1.2	1.2
Depression or anxiety/tension/sleep problem	0.9	1.2	0.8	1.0
Other	14.0	9.2	7.7	8.4

### Treatment outcome of last outpatient visit

Among three different health care agencies, the AYUSH practitioner had the highest positive (got better or much better) treatment outcome (85.6%), followed by the biomedical practitioner (85.1%) and traditional healer (82.9%). Similarly, 88.7% of AYUSH practitioner patients were satisfied or very satisfied with their last outpatient visit, 86.3% of biomedical, and 79.3% of traditional healer patients. The mean expenditure on the health care providers fees of the last outpatient visit was the highest for the biomedical practitioner (227 Rs), followed by the traditional health (152 Rs) and the AYUSH practitioner (150 Rs), while majority of THP patients had no expenditure (62.6%) and the biomedical practitioner (35.5%) and AYUSH practitioner (35.4%) (see Table 4).

**Table 4:** Treatment outcome of last outpatient visit (N = 37,852)

Variable	Biomedical practitioner	AYUSH practitioner	Traditional healer
	%	%	%
*Outcome of most recent visit to the health care provider*			
Got much better	11.8	14.6	13.6
Got better	73.3	71.0	69.3
Had no change	14.2	13.9	16.8
Got worse	0.5	0.3	0.2
Got much worse	0.1	0.1	0.1
*Satisfaction with health care received during last outpatient visit*			
Very satisfied	13.9	17.6	12.4
Satisfied	72.4	71.1	66.9
Neither satisfied nor dissatisfied	12.3	10.6	19.1
Dissatisfied	1.3	0.5	1.5
Very dissatisfied	0.1	0.2	0.1
No expenditure on health care providers fees in last out-patient visit	35.5	35.4	62.6
Mean expenditure on health care providers fees in last out-patient visit (Rs)	M = 227 (SD = 586)	M = 150 (SD = 385)	M = 152 (SD = 317)

### Associations with AYUSH and traditional health practitioner utilization

In the adjusted logistic regression analysis, older age (≥60 years) (Adjusted Odds Ratio-AOR: 1.20, 95% Confidence Interval-CI: 1.07–1.34), having pain (AOR: 1.48, 95% CI: 1.29–1.69), any bone or joint diseases (AOR: 1.57, 95% CI: 1.35–1.82) and current tobacco use (AOR: 1.30, 95% CI: 1.12–1.50) were positively and male sex (AOR: 0.76, 95% CI: 0.68–0.85), high subjective socioeconomic status (AOR: 0.72, 95% CI: 0.60–0.87), urban residence (AOR: 0.71, 95% CI: 0.57–0.88), diabetes (AOR: 0.66, 95% CI: 0.55–0.81), chronic heart disease (AOR: 0.52, 95% CI: 0.37–0.73), and having a health insurance cover (AOR: 0.36, 95% CI: 0.30–0.44) were negatively associated with AYUSH practitioner utilization in the past 12 months. In a second adjusted logistic regression analysis, depressive symptoms (AOR: 1.17, 95% CI: 1.01–1.35), sleep problems (AOR: 1.28, 95% CI: 1.08–1.51), having pain (AOR: 1.82, 95% CI: 1.55–2.15), and current tobacco use (AOR: 1.35, 95% CI: 1.22–1.51) were positively associated with past 12-month traditional healer utilization. Furthermore, having health insurance cover (AOR: 0.41, 95% CI: 0.33–0.51), hypertension (AOR: 0.82, 95% CI: 0.71–0.95), diabetes (AOR: 0.50, 95% CI: 0.39–0.65), urban residence (AOR: 0.25, 95% CI: 0.19–0.34), and high subjective socioeconomic status (AOR: 0.70, 95% CI: 0.58–0.85), were negatively associated with past 12-month traditional healer utilization. (see Table 5).

**Table 5:** Associations with past-12-month AYUSH and traditional health practitioner use

Variable	AYUSH practitioner use		Traditional/Folk healer use	
	Crude Odds Ratio (95% CI)	Adjusted Odds Ratio (95% CI)	Crude Odds Ratio (95% CI)	Adjusted Odds Ratio (95% CI)
Age (years)				
45–59 (female spouse: any age)	1 (Reference)	1 (Reference)	1 (Reference)	1 (Reference)
≥ 60	1.22 (1.09, 1.35)***	1.20 (1.07, 1.34)**	1.13 (1.01, 1.28)*	0.95 (0.83, 1.08)
Sex				
Female	1 (Reference)	1 (Reference)	1 (Reference)	–
Male	0.85 (0.78, 0.93)***	0.76 (0.68, 0.85)***	0.95 (0.88, 1.03)	
Subjective socioeconomic status				
Low (1–3)	1 (Reference)	1 (Reference)	1 (Reference)	1 (Reference)
Medium (4–5)	0.89 (0.77, 1.02)	0.95 (0.82, 1.11)	0.63 (0.55, 0.72)***	0.74 (0.65, 0.84)***
High (6–10)	0.62 (0.51, 0.76)***	0.72 (0.60, 0.87)***	0.51 (0.41, 0.63)***	0.70 (0.58, 0.85)***
Residence				
Rural	1 (Reference)	1 (Reference)	1 (Reference)	1 (Reference)
Urban	0.61 (0.48, 0.77)***	0.71 (0.57, 0.88)**	0.17 (0.12, 0.23)***	0.25 (0.19, 0.34)***
Hypertension	0.85 (0.75, 0.96)**	0.91 (0.80, 1.04)	0.64 (0.55, 0.73)***	0.82 (0.71, 0.95)**
Diabetes	0.57 (0.47, 0.69)***	0.66 (0.55, 0.81)***	0.32 (0.25, 0.41)***	0.50 (0.39, 0.65)***
Chronic lung disease	1.14 (0.92, 1.40)	–	0.83 (0.67, 1.03)	–
Cancer	1.28 (0.76, 2.14)	–	1.14 (0.51, 2.55)	–
Chronic heart disease	0.49 (0.36, 0.68)***	0.52 (0.37, 0.73)***	0.51 (0.32, 0.81)**	0.68 (0.43, 1.09)
Stroke	1.11 (0.81, 1.53)	–	0.74 (0.52, 1.05)	–
Any bone or joint diseases	1.57 (1.34, 1.83)***	1.57 (1.35, 1.82)***	1.02 (0.88, 1.18)	–
Neurological or psychiatric problem	0.93 (0.68, 1.27)	–	0.81 (0.58, 1.13)	–
Depressive symptoms	1.13 (0.97, 1.33)	–	1.39 1.19, 1.62)***	1.17 (1.01, 1.35)*
Sleep problems	1.01 (0.86, 1.19)	–	1.57 (1.31, 1.88)***	1.28 (1.08, 1.51)**
Symptom-based pain	1.72 (1.49, 1.97)***	1.48 (1.29, 1.69)***	2.10 (1.76, 2.51)***	1.82 (1.55, 2.15)***
Current tobacco use	1.26 (1.11, 1.44)***	1.30 (1.12, 1.50)***	1.62 (1.44, 1.82)***	1.35 (1.22, 1.51)***
Has health insurance	0.38 (0.31, 0.46)***	0.36 (0.30, 0.44)***	0.43 (0.34, 0.53)***	0.41 (0.33, 0.51)***

*CI* Confidence Interval; ***p < 0.001;**p < 0.01; *p < 0.05

## Discussion

The study found that in a large national middle-aged and older adult sample in India in 2017–2018, the prevalence of past 12-month AYUSH or THP utilization was 13.0% (6.5% AYUSH and 7.0% THP). The prevalence of AYUSH utilization in the past 12 months (6.5%) is similar to a previous national survey in India in 2014 (6.9% for all ages and 7.7% for persons 60 years and older of the past 2 weeks health care provider visit) [18], and the prevalence of AYUSH and/or THP utilization (13.0%) was lower than in a national study among persons predominantly 50 years and older in India in 2007 (19.0% THP use in the previous 12 months) [5], and higher than in a small study among older adults (≥60 years) in Maharashtra, India (3.6%) [20]. Using the same reference period (12 months), this survey showed lower utilization rates than in a previous review (21.1% TCM provider use) [9] and in a 32-country study (26.4% TCM provider use) [10]. In a national survey in Indonesia, 5.6% of participants (15 years and older) had utilized a TCM practitioner in the past 4 weeks [28], while in our survey the past 12-month prevalence of AYUSH and/or THP use was 13.0%. The prevalence of AYUSH and/or traditional health practitioner use varied by state in India. Some states had a high utilization rate while some had no utilization. For example, the highest prevalence of AYUSH practitioner utilization in the past 12 months was in Uttar Pradesh (18.7%), followed by Maharashtra (13.8%), Bihar (8.1%), Kerala (7.7%) and West Bengal 6.9%), which is in some cases similar to a previous survey in India, with high rates of past 2 weeks AYUSH utilization in Kerala (13.7%) and West Bengal (11.6%), but lower rates in Uttar Pradesh (8.6%) and Maharashtra (4.0%) [18]. Some of these regional differences may be attributed to the availability of AYUSH practitioners, which highly differs by state in India. For example, among all states, the third highest number of Ayurveda registered practitioners in 2010 was in Uttar Pradesh (53735) [29], the highest number of UNANI practitioners (16967) [30], the highest number of homeopathy practitioners in 2010 was in Uttar Pradesh (1575) [31]. However, Uttar Pradesh is also India’s most populous state (199 million) [32]. The prevalence of THP utilization was the highest in Bihar (24.3%). This result seems to be consistent with a previous study among the ethnic communities in Bihar, where primarily THP do the treatment of different ailments [33], and many people “continue to depend on local medicinal plants at least for the treatment of primary healthcare” [34].

In agreement with some previous studies [18, 35–41], this study found that in older age (≥60 years) was associated with AYUSH and in in unadjusted analysis with THP utilization. Several studies showed that female sex was associated with TCM provider use [18, 35, 40], which in this study was found for AYUSH utilization but not for traditional health practitioner utilization. This investigation found that lower socioeconomic status increased the odds of AYUSH and THP utilization. Similar results were found in two studies among older adults in India [19, 20], while some other studies found mixed results [18, 28, 35, 40]. Reasons for the higher utilization of AYUSH and THP among persons with lower socioeconomic status may be attributed to the lower costs of AYUSH practitioner and THP consultation than biomedical care, as found in this study. Most previous studies [35–38] found an association between rural residence and TCM use, which is confirmed in this study for both AYUSH and THP utilization. Reasons for this may be attributed to subsidized public health care mostly serving economically better-off urban dwellers, while rural public health care is poorly staffed and has poorer quality, increasing the dependency on private providers, such as AYUSH and THP, for health care [18].

In concordance with previous studies [19, 28, 41, 42], this study found that having a chronic condition (any bone or joint diseases and chronic pain) [19, 42], and tobacco use [41], were associated with both AYUSH and THP utilization, while sleep problems [28] and depressive symptoms [19, 28, 42] were associated with THP utilization. Furthermore, the study showed having other chronic conditions (hypertension, diabetes and chronic heart disease) decreased the odds of AYUSH and/or THP utilization. It is possible that middle-aged and older Indians believe that THP are less effective in treating chronic diseases, such as hypertension, diabetes and chronic heart diseases than the biomedical care system. The positive association between tobacco use and AYUSH and THP use may be explained by the increased likelihood of suffering from health problems requiring treatment, including TCM.

In this study, among the conditions treated at the most recent outpatient visit, the highest prevalence of AYUSH and/or THP use was found for fever or pyrexia of unknown cause (37.6%), followed by chronic pain (17.7%), generalized pain (12.7%), and high blood pressure (4.6%). Pain and fever are commonly treated by THP in India [43–45] and AYUSH practitioners [46, 47]. In a study in 18 states in India the highest number of cases treated by AYUSH included acute problems such as “cold-cough-fever, diarrhoea, digestive disorders such as abdominal pain/indigestion/vomiting/gas/acidity, and difficulty in breathing.” [2]. In a previous survey in India, AYUSH utilization was high for skin diseases [18] and musculoskeletal related diseases [18, 20], which was similar for the treatment of chronic pain in joints, back, neck or muscle at the second highest prevalence and at a lower prevalence for skin diseases in this study. Regarding high blood pressure, in community-based surveys in several Southeast Asian countries, the use of TCM for hypertension was 1.9% in Laos [48], 7.2% in Indonesia [28], 13.4% in Myanmar and 15.3% in Cambodia [48]. The prevalence using AYUSH and THP for malaria was about 1.2%, similarly to the biomedical sector (1.2%). In a qualitative study in the district of Gadchiroli, Maharashtra state in India, widespread misconceptions about malaria and treatment by unqualified traditional healers delayed effective treatment seeking [49]. The higher utilization of AYUSH and/or THP for fever or pyrexia of unknown cause, chronic pain in joints, arthritis, rheumatism or osteoporosis, generalized pain (stomach, headache, migraine, or other nonspecific pain) than allopathic health care services, may emphasise their role in the provision of both acute and chronic care services. From a policy perspective AYUSH and THP services may be expanded to include both acute and chronic care [2].

In this study, satisfaction with the last health care visit with an AYUSH and THP were (88.7 and 79.3%, respectively, were satisfied or very satisfied). Results compare with 90% satisfaction with TCM among adults in Lebanon [39], and 85.7% satisfaction with the last THP visit among adults in Indonesia [28]. Majority rated their treatment outcome as positive (got better or much better), 85.6% for the AYUSH practitioner and 82.9% for the THP in this study, which compares 80% of very or somewhat helpful TCM provider consultations among adults with chronic conditions in lower Mekong countries [35]. Similarly, to a previous national survey in India in 2001–2002, the mean expenditure on allopathic consultation was higher (Rs 39 in 2001–2 and 227 Rs in 2017–18) than for AYUSH consultation (Rs 29 in 2001–2 and 150 Rs in 2017–18) [50]. In addition, 62.6% of THP patients had no expenditure on consultation, but only 35.4% with the AYUSH practitioner and 35.5% with the biomedical practitioner. Furthermore, in this study, having no health insurance cover was associated with both AYUSH and THP utilization, which compares with previous results in a study in Lebanon [39]. Individuals having a health insurance cover were less likely utilizing AYUSH and THP, meaning that patients may more likely consult THP because of less or no expenditure.

A significant proportion of the general middle-aged and older adult population in India consult AYUSH and/or THP. While AYUSH has been integrated into health care services and follows some accreditation and certification, this is not the case for THP. Considering the importance of THP, “massive documentation and validation of the local heath practices by the AYUSH context specific epistemology and the linkage between the two should be undertaken by the district and state level bodies for promotion and use.” [2].

There could be a risk for interactions between TCM, in particular THP, and biomedical medicine, calling for the need for TCM and THP medicines and providers to be regulated [18, 51]. Health care providers should educate patients on TCM, in particular THP, use and on combining the use of TCM and biomedical medicine, with a particular focus on less educated persons and those with sleep problems, depressive symptoms, chronic pain and tobacco use.

### Study strength and limitations

The strength of the study was a nationally representative sample of middle-aged and older adults in India and the use of standardized measures adapted from the Health and Retirement Study. The self-report of most data may have their limitations. It possible that participants underreported the utilization of AYUSH and THP. Furthermore, this study was based on cross-sectional data, and we therefore cannot ascribe causality to any of the associated factors in the study. Some questions asked about over-the-counter medicine use, including AYUSH and traditional and complementary medicine, but since they were only asked for a subsample of the survey, we excluded them from the results. More details regarding the type of AYUSH and traditional medicines and for which ailments could have been assessed and included in future research.

## Conclusions

The study confirms a moderate prevalence of AYUSH and THP utilization in India. Sociodemographic and health-related factors such as older age, female sex, lower socioeconomic status, rural residence, not having hypertension, not having diabetes, not having chronic heart disease, pain, any bone or joint diseases, tobacco use, sleep problems and depressive symptoms, and not having health insurance were found to be associated with utilization of AYUSH and/or THP.

## Data Availability

The data are available at the Gateway to Global Aging Data (https://g2aging.org/?section=overviews&study=lasi).

## Abbreviations

ADL: Activities of Daily Living
AYUSH: Ayurveda/Yoga/Naturopathy/Unani/Siddha /Homeopathy
BMI: Body Mass Index
IADL: Instrumental Activities of Daily Living
LASI: Longitudinal Ageing Study in India
LHT: Local Health Traditions
TCM: Traditional and Complementary Medicine
THP: Traditional health practitioner
